# Secular Trends of Gestational Diabetes Mellitus and Changes in Its Risk Factors

**DOI:** 10.1371/journal.pone.0136017

**Published:** 2015-08-20

**Authors:** Geum Joon Cho, Log Young Kim, Ye Na Sung, Jee Ae Kim, Soon Young Hwang, Hye-Ri Hong, Soon-Cheol Hong, Min-Jeong Oh, Hai-Joong Kim

**Affiliations:** 1 Department of Obstetrics and Gynecology, College of Medicine, Korea University, Seoul, Korea; 2 The Health Insurance Review & Assessment Service of Korea, Seoul, Korea; 3 Department of Biostatistics, College of Medicine, Korea University, Seoul, Korea; 4 Department of Obstetrics and Gynecology, School of Medicine, Kyung Hee University, Seoul, Korea; Virgen Macarena University Hospital, School of Medicine, University of Seville, SPAIN

## Abstract

**Objective:**

The aim of this study was to evaluate the secular trends of incidence of gestational diabetes mellitus (GDM) and insulin treatment for GDM in a Korean population and to determine the factors that contribute to the trends in the incidence of GDM.

**Study Design:**

We used data collected by the Health Insurance Review & Assessment Service of Korea and analyzed data from women who had given birth from 2006 to 2010. We evaluated the trends in the incidence of GDM and GDM requiring insulin treatment and the changes in risk factors.

**Results:**

There were 1,824,913 births during the study period, which included 129,666 cases of GDM, an incidence of 7.11% over this period. The incidence of GDM increased from 3.86% in 2007 to 11.83% in 2010, with a continuous increase after adjustment for age. However, the number of GDM cases that required insulin treatment decreased significantly from 13.87% in 2007 to 5.94% in 2010. The proportion of patients who were at an older age and multiparity, 2 GDM risk factors, increased during the study period.

**Conclusions:**

In Korea, the incidence of GDM, especially mild GDM, increased dramatically during the period from 2006 to 2010. Further efforts are needed to monitor this trend and to identify associated factors.

## Introduction

Gestational diabetes mellitus (GDM) is defined as carbohydrate intolerance with its onset or diagnosis during pregnancy [1]. Although GDM is not the only contributor to maternal and neonatal morbidity, it accounts for a significant proportion of the global burden of disease [2]. The incidence of GDM varies from 2.4% to 22.3% worldwide depending on the population and the type of diagnostic test employed [3, 4]. Several studies have examined the trend in the incidence of GDM among pregnant women in various populations, and an increase in incidence has been generally observed [1, 5–11].

It is essential to determine the cause of increase in the incidence of GDM not only to better understand trends, but to also identify modifiable risk factors that may be addressed, in order to prevent the onset of GDM [1, 5]. GDM is associated with pregnancies occurring at an older age, higher parity, higher pre-pregnancy weight and BMI, a history of diabetes in first-degree relatives, a past history of gestational diabetes, and Asian ethnicity [8–10, 12–15]. Several studies have evaluated factors that increase the incidence of GDM and have reported older maternal age, increasing obesity, and diabetes may increase the incidence of GDM [1, 7–10]. Although Asian ethnicity is one of the most important risk factors in the development of GDM, there is limited information about trends in the incidence of GDM for the Asian population and factors that contribute to the changes in these trends[1, 8, 11].

GDM is classified into 2 subcategories: A1 and A2 [16]. Class A1 GDM corresponds to carbohydrate intolerance during an oral glucose tolerance test (OGTT), but fasting and postprandial glucose levels are maintained within the physiologic range using only dietary regulation. Class A2 includes gestational diabetic women who require insulin. This differentiation is important because women with GDM who require insulin treatment are at a greater risk for perinatal complications than those whose diabetes can be controlled by diet alone [17]. Moreover, insulin treatment during pregnancy is the strongest predictor for the long-term development of type 2 diabetes [18]. However, the definition of “gestational diabetes” used in various studies investigating the trends of GDM fails to specify whether the patient requires dietary regulation alone or treatment with dietary regulation and insulin.

Therefore, the aim of this study was to evaluate the secular trends in the incidence of GDM and GDM requiring insulin treatment in a Korean population and to determine the factors that contribute to the trends in GDM incidence.

## Materials and Methods

This study was conducted using 2 different data sets. First, study data of GDM incidence and its risk factors in pregnant women were collected from the Korea National Health Insurance (KNHI) Claims Database of the Health Insurance Review and Assessment Service (HIRA) for 2007–2010. In Korea, 97% of the population is enrolled in the obligatory KNHI program. Healthcare providers are required, according to health insurance policies, to allow HIRA to review the medical costs incurred during the treatment of patients. The remaining 3% of the population are covered by the Medical Aid Program. Thus, the HIRA database contains information on all claims for approximately 50 million Koreans, and nearly all information about disease incidence can be obtained from this centralized database, with the exception of procedures that are not covered by insurance, such as cosmetic surgery. According to the Act on the Protection of Personal Information Maintained by Public Agencies, HIRA prepares claims data by concealing individual identities. Therefore, studies using data from HIRA can be exempt from institutional board reviews [19]. Data we received included an unidentifiable code representing each individual’s age, diagnosis, and a list of prescribed procedures. The International Classification of Diseases, tenth Revision (ICD-10) diagnosis and procedure codes were used to identify all women who had given birth during 2007–2010. To identify patients with GDM from the HIRA database, we used ICD-10 codes O24.4 and O24.9. Data regarding the prescription of insulin were analyzed. In this study, the type of insulin that was administered was not distinguished. To identify risk factors for GDM, data of the women’s characteristics such as age, multiple pregnancies (defined as twin or higher-order gestation), and multiparity were obtained. Maternal age was categorized as <25, 25–29, 30–34, 35–39, and ≥40 years.

In accordance with the recommendation of the American College of Obstetricians and Gynecologists [20], The Korea Society of Obstetrics and Gynecology recommends that risk assessment for all pregnant women should be performed at the first prenatal visit. High risk women should undergo screening as soon as possible. If negative at first visit, high risk women should be retested at 24–28 wks. Women who are not at high risk for GDM should have screening at 24–28 wks. Screening can be done as 50-g glucose challenge test followed by diagnostic 100-g, 3hour oral glucose tolerance test if abnormal (two-step approach). A diagnosis of GDM is made when two or more glucose vales fall at or above the glucose thresholds proposed by either Carpenter and Coustan (CC) criteria or the National Diabetes Data Group (NDDG) criteria.

Second, study data of the risk factors for GDM in women of child-bearing age were collected from the National Health and Nutrition Examination Surveys (NHANES) IV (2007–2009) and V (2010). KNHANES is a nationwide, population-based, and cross-sectional designed health survey conducted by the Division of Health and Nutritional Survey under the Korean Centers for Disease Control and Prevention [21]. In brief, the survey consists of 3 components: a health interview survey, nutrition survey, and health examination survey. Participants were selected from populations based on geographical area, sex, and age using household registries with a stratified, multistage, clustered, probability sampling design. This sampling method is certified as producing representative statistics by the Korea Department of Statistics. A total of 33,829 participants aged >1 yr responded to the health questionnaire. After exclusion of participants who had ≥1 missing value related to GDM, 2,174 women aged 19–44 years (280 in 2007, 600 in 2008, 705 in 2009, and 589 in 2010) were included in this study.

The health interview survey included questions regarding demographic, socioeconomic, and lifestyle status. Data for the following covariates were obtained using a standardized questionnaire in the KNHANES: age, smoking history, and exercise level. Smoking history was divided into 3 categories: current smoker, past smoker, and never a smoker, based on the answer to “Have you ever been a smoker?” and “If yes, do you smoke currently?” Moderate exercise was defined as >30 min of moderate physical activity, in which the subject was tired compared with that at ordinary resting level or performed activities resulting in slightly higher breathing for more than 5 days per week.

Body mass index ([BMI] in kilograms per square meter) was calculated using height and weight measurements. Waist circumference (WC) was measured from the narrowest point between the borders of the rib cage and the iliac crest with the patient at rest. Obesity was defined as BMI ≥ 25 kg/m2, which was adopted from the cutoffs established for Korean adults as proposed by the Korean Society for the Study of Obesity [22]. Abdominal obesity was defined as a waist circumference ≥85 cm according to the Korean Society for the Study of Obesity [23]. Blood pressure (BP) was measured using a standard mercury sphygmomanometer. Hypertension was defined as systolic/diastolic BP ≥ 140/90 mm Hg or if the patient was taking antihypertensive medication. Blood samples were obtained after a minimal fast of 8 hours. The levels of high-density lipoprotein cholesterol (HDL-C), triglycerides (TG), and fasting glucose were measured with a Hitachi 747 autoanalyzer (Hitachi Instruments Inc., Tokyo, Japan) by using enzymatic methods. Low HDL-C level (<50 mg/dL for women) and high TG level (≥150 mg/dL) were defined using the National Cholesterol Education Program Adult Treatment Panel III criteria [24]. Criteria for high glucose level (fasting glucose level ≥100 mg/dL, or currently being treated for diabetes) were adopted from the guidelines established by the American Diabetes Association [25]. Diabetes mellitus (DM) was defined as having a fasting glucose level ≥126 mg/dL or currently being treated for diabetes. Unrecognized DM was defined as newly diagnosed DM, after the exclusion of patients with previously known DM (patients taking oral antidiabetic agents or insulin, or patients who were following a diet prescribed by a physician).

The 24-hour dietary recall method was used to collect data regarding food items consumed by the study participants during a 24-hour period. The energy and fat intake derived from the consumption of each food item was calculated.

## Statistics

The incidence for GDM by year and by age group was calculated using the HIRA data. The time trend of GDM during 2007–2010 was tested using an analysis of covariance after adjusting for age. For each age group, the Cochran-Armitage trend test was performed to identify trends by year. In the study population, the mean with standard deviations or the percentage and the tested trend were calculated in order to compare the basic characteristics related to risk factors by year. Risk factors associated with GDM were analyzed using multivariate logistic regression analysis.

For the KNHANES data, sampling weights were used to calculate all estimates for factors associated with GDM in women of child-bearing age. The proportions and standard errors, or the means and standard errors by year were estimated, and the time trends by year were tested using the analysis of covariance for continuous variables and the multiple logistic regression analysis for categorical variables. Statistical analysis was conducted using SAS 9.3 (SAS Institute, Inc, Cary, NC). P <0.05 was considered statistically significant.

## Results

There were 1,824,913 birth recorded during the study period, which included 129,666 cases of GDM, an incidence of 7.11%. Among GDM cases, the overall incidence of GDM requiring insulin treatment was 8.61% (11,160/129,666).

**Fig 1:**
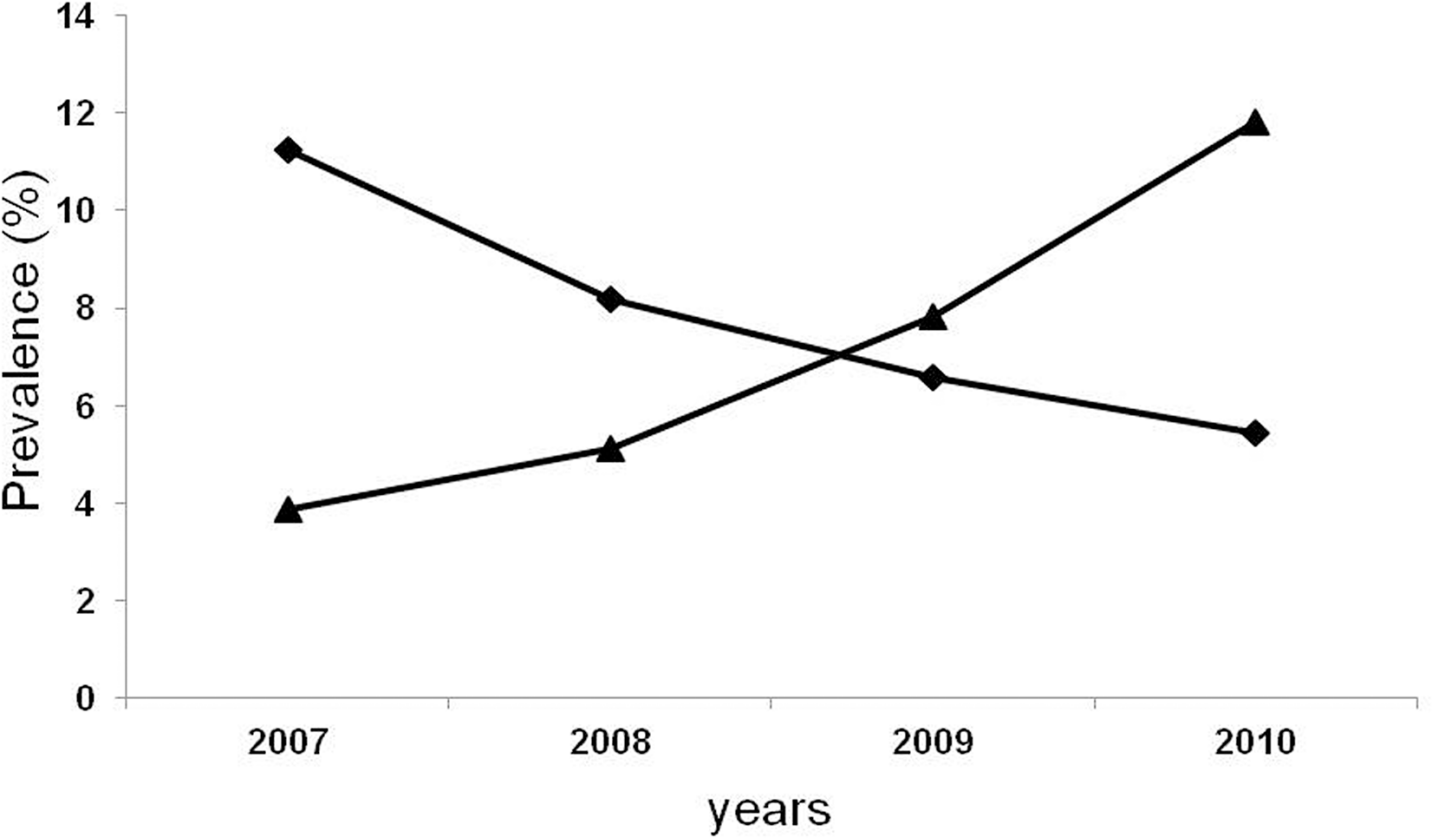
The trend for GDM prevalence (▲) and GDM with insulin treatment among all GDM (◆).

The incidence of GDM increased from 3.86% in 2007 to 11.83% in 2010, with a continuous increase observed after adjustment for age (P < 0.001; Fig 1), but the proportions of GDM requiring insulin treatment among GDM decreased significantly from 13.87% in 2007 to 5.94% in 2010 (P < 0.001; Fig 1).

**Fig 2:**
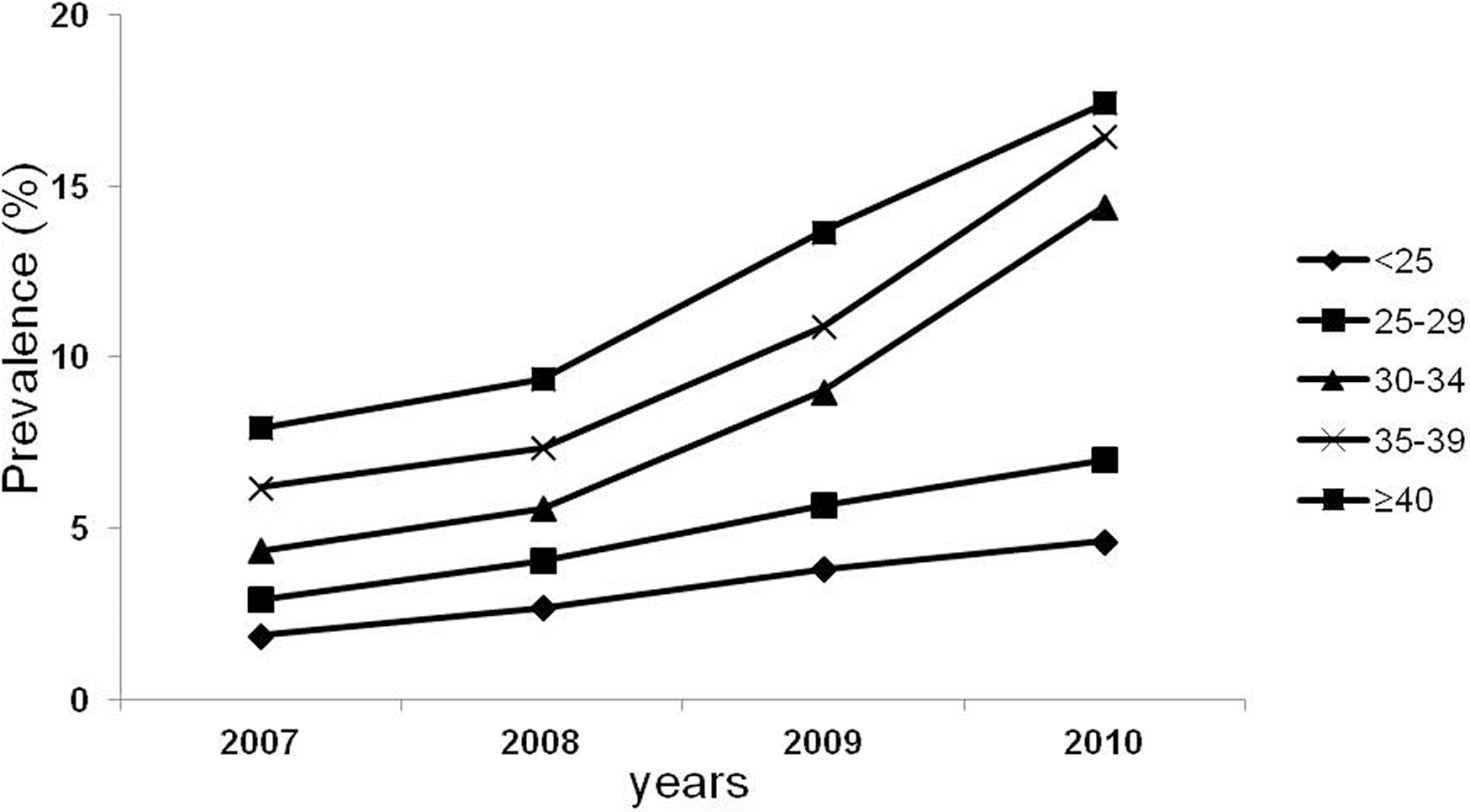
The trend for GDM prevalence by age group.

The incidence of GDM increased in all age groups with the highest incidence in the 30–34 age group (Fig 2).

The trends of risk factors for GDM are summarized in Table 1. The proportion of pregnancies at an older maternal age and multiparity were increased, but the proportions of multiple pregnancies significantly decreased during the study period.

**Table 1:** The basic characteristics of the study population (2007–2010).

	2007	2008	2009	2010	p value for trends
Age (years)	30.04 ± 3.93	30.26 ± 3.97	30.44 ± 3.98	30.73 ± 3.99	<0.001
Older maternal age (≥35 years)(%)	12.56	13.96	15.03	16.66	<0.001
Multiparity (%)	46.52	46.56	46.77	48.37	<0.001
Multiple pregnancies (%)	1.43	1.41	1.40	1.38	<0.001

Data are mean±standard deviation or %.

Multivariate-adjusted odds ratios (ORs) for GDM are summarized in Table 2. Pregnant women at 35 years of age and older and those with multiparity, and multiple pregnancies had an increased risk of GDM.

**Table 2:** The adjusted ORs for the risk of GDM.

	OR^a^	95% CI
Age (≥35)	2.762	(2.615, 2.918)
Multiparity	1.078	(1.024, 1.135)
Multiple pregnancy	2.262	(1.967, 2.602)

^a^ORs were adjusted for all variables in the table.

The secular changes in factors related to GDM in women of child-bearing age are summarized in Table 3. Among various risk factors, energy and fat intake increased during the study period, but WC decreased. However, there were no changes in the rate of other factors, including smoking and exercise, BMI, HDL, TG, LDL, and fasting glucose levels, and the incidence of DM and unrecognized DM during the study period.

**Table 3:** Secular trends of risk factors for GDM among women of child-bearing age from 2007 to 2010.

	2007 (n = 280)	2008 (n = 600)	2009 (n = 705)	2010 (n = 589)	P-value for trends
Currently smokes (%)	10.41 ± 1.47	13.93 ± 1.13	14.23 ± 1.11	7.30 ± 0.93	0.116
Moderate exercise (%)	7.35 ± 1.17	12.83 ± 1.11	11.64 ± 0.84	8.65 ± 0.91	0.566
BMI (kg/m^2^)	22.09 ± 0.12	22.34 ± 0.10	22.39 ± 0.12	22.16 ± 0.12	0.642
*Obesity by BMI (%)	14.93 ± 1.41	17.87 ± 1.19	18.92 ± 1.12	17.40 ± 1.26	0.170
WC (cm)	75.64 ± 0.44	75.16±0.35	74.42±0.34	73.73 ± 0.34	<0.001
†Obesity by WC (%)	13.35 ± 1.46	14.29 ± 1.08	13.32 ± 0.94	11.39 ± 0.99	0.210
HTN (%)	2.55 ± 0.75	4.34 ± 0.56	5.97 ± 0.71	3.69 ± 0.51	0.125
HDL (mg/dL)	52.38 ± 0.48	53.08 ± 0.37	52.29 ± 0.34	52.72 ± 0.33	0.835
Low HDL (%)	46.71 ± 2.26	40.97 ± 1.48	44.75 ± 1.37	40.70 ± 1.49	0.071
TG (mg/dL)	93.51 ± 2.25	92.95 ± 2.34	91.87 ± 1.99	89.57 ± 1.96	0.154
High TG (%)	12.98 ± 1.21	11.31 ± 0.82	12.27 ± 0.92	10.51 ± 0.99	0.163
Fasting glucose (mg/dL)	87.61 ± 0.56	91.56 ± 0.58	90.35 ± 0.44	89.17 ± 0.44	0.131
High glucose (%)	6.54 ± 0.94	11.23 ± 0.80	10.42 ± 0.94	8.00 ± 0.86	0.401
Energy intake (kcal)	1637.08 ± 27.81	1660.77 ± 18.84	1666.40 ± 18.34	1807.86 ± 22.36	<0.001
Fat intake (g)	36.06 ± 1.07	36.55 ± 0.69	37.67 ± 0.69	42.87 ± 0.81	<0.001
DM (%)	1.37 ± 0.42	2.14 ± 0.37	2.30 ± 0.41	2.03 ± 0.55	0.346
Unrecognized DM (%)	0.57 ± 0.27	1.00 ± 0.27	0.70 ± 0.24	0.52 ± 0.26	0.667

Data are mean±standard errors or %±standard errors. BMI, body mass index; WC, waist circumference; HTN, hypertension; HDL-C, high-density lipoprotein cholesterol; TG, triglyceride; DM, diabetes mellitus.

*Obesity was defined as BMI ≥25 kg/m^2^.

†Obesity was defined as WC≥85cm.

## Discussion

In this study, the incidence of GDM increased sharply during the 4-year study period. These results are consistent with those from other studies [1, 6–10]. Although the reason for this trend is unclear, there are several possible explanations. First, multiple factors likely influence the trend in the incidence of GDM, and the observed trend may reflect complex changes in the rate of risk factors. In this study, women who were older during their pregnancy were at a significant risk for developing GDM, consistent with results from previous studies [9, 10]. As expected, we found that the rate of older maternal age increased during the study period. This risk factor likely contributed to the observed increase in the overall incidence of GDM, which was in agreement with results from other studies [9]. Nonetheless, we found that the incidence of GDM increased for all age groups; moreover, after adjustment for age, the incidence of GDM increased indicating that factors than other older maternal age may be contributing to the increased incidence of GDM, indicating that the observed increase in GDM incidence may be independent of age [7, 8].

Ferrara et al. suggested that the increased incidence of GDM might reflect or contribute to the ongoing pattern of increasing obesity and diabetes [1]. Obesity is one of the strongest risk factors of GDM [12], and its incidence has been dramatically increasing over the last several decades [5]. However, due to lack of data, little is known about the association between the increase in GDM incidence and concomitant increases in maternal obesity [1]. The results from a study examining the incidence of pre-pregnancy obesity are inconsistent. In the US, the incidence of pre-pregnancy obesity increased by about 70% between 1993 and 2003 [26]. However, other studies reported that pre-pregnancy obesity did not increase significantly between 2000 and 2009 [5], and the temporal increase in maternal glucose disorders over the 10-year period could not be explained by similar increases in BMI [5]. In this study, the incidence of obesity based on BMI in women of child-bearing age did not change, but that based on WC decreased significantly, which is in agreement with the results from hospital-based studies reporting no changes in pre-pregnancy BMI of pregnant Korean women during the past 2 decades [27, 28]. These discrepancies between the studies may be due to the specifics of obesity epidemics; pre-pregnancy obesity especially varies according to age, region, ethnicity, and population, and is dynamically changing with time [29]. In this study, therefore, obesity may have minimal effects on the increase in the incidence of GDM.

Several studies based on national data reported an increase in pre-pregnancy diabetes [6]. GDM is defined as carbohydrate intolerance with onset or diagnosis during pregnancy [1]. Therefore, the observed trend in GDM may be because of increased diabetes incidence, especially unrecognized diabetes existing before pregnancy. However, the incidence of diabetes and unrecognized diabetes did not change during the study period.

Physical inactivity [30], diets high in saturated fat [31], and smoking [32] are associated with an increased risk for GDM. Among various risk factors, intake of calories and fat increased during the study period. Park et al. reported that high energy and saturated fat intakes were common risk factors for GDM and pregnancy outcome such as large-for-gestational age infants in Korea [33]. Therefore, these may partially contribute to an increase in the incidence of GDM. However, other factors, including physical inactivity, smoking status, and lipid profiles, were not changed.

Another factor that may have influenced trends in GDM incidence in our study is changes in screening recommendations. The observed trends in this study may be influenced by improvements in screening over the study period [10]. However, several studies reported that increased incidence of GDM was not caused by the increased proportion of screened pregnancies [6, 7]. Universal screening for GDM is recommended in Korea, although there are no published reports regarding the actual screening data. We believe that there have been minor improvements in screening over time and minor effects on the increase in the incidence of GDM.

The trends observed in this study may be attributed to changes in diagnostic criteria. Both the Fourth International Workshop-Conference on GDM and the American Diabetes Association have endorsed CC criteria [34, 35]. Compared with NDDG criteria, the use of more inclusive criteria enabled an increased diagnosis of GDM by 30–50%, with varying magnitude depending on maternal age and race or ethnicity [36–38]. The Korean Society of Obstetrics and Gynecology recommends using either CC criteria or NDDG criteria when diagnosing GDM. However, there is no information about the criteria that are generally used by practitioners in Korea. Thus, it is difficult to evaluate the shift in criteria between CC criteria and NDDG criteria during study period and its effects on trends of GDM incidence. Although we believe that there have been minor shift in criteria from NDDG to CC criteria over time and minor effects on the increase in the incidence of GDM, further studies are needed to evaluate the effect of the criteria used by practitioners in diagnosing GDM and their contribution to the increase in the incidence of GDM.

In our study, approximately 8.61% of women with GDM required insulin treatment. This is substantially lower than the previously reported 20–50% of women who required insulin treatment [39, 40]. This may be owing to ethnic differences in the clinical characteristics of patients with GDM, with Southeast Asian women having the lowest need for insulin treatment [41] and the study population enrolled in our study being based on a nation-wide population, but other studies being based on tertiary hospital-patients [39, 40]. Specific characteristics in the increase in the incidence of GDM may also contribute to the wide difference in the reports of the percentage of women with GDM requiring insulin treatment. This is the first large population study to evaluate changes in the incidence of GDM requiring insulin treatment. Although increasing trends in GDM patients requiring insulin treatment were observed, overall, the proportion of GDM cases requiring insulin treatment decreased sharply. These results imply that the increased incidence of GDM observed in this study was owing to a dramatic increase in GDM cases requiring only nutritional treatment, i.e., mild GDM, which is in contrast to the results of a study that reported that the increased incidence of GDM was not caused by an increase in the proportion of pregnancies complicated by milder hyperglycemia [7]. Therefore, the lower incidence of GDM patients requiring insulin treatment may be due to a disproportionate increase in the number of mild GDM cases.

Several limitations should be kept in mind when interpreting our findings. First, the incidence of GDM in this study was based on insurance claims data from the KNHI Claims Database, which was designed for cost claim issues and not for research purposes. Thus, the main limitation is the validity of data in this database. However, the KNHI data have been validated in a previous study [42]. Another limitation of our study is that we were not able to access information such as maternal BMI, socio-demographic factors, family history of DM, previous assisted reproductive technique and laboratory test results, all of which are factors related to GDM, because these data were not available in the database. Thus, we could not investigate changes in risk factors related to GDM in pregnant women and could only indirectly evaluate trends in this population using the KNANES data. Further studies are needed to investigate changes in risk factors related to the incidence of GDM in pregnant women. Nevertheless, the strength of the present study lies in the evaluation of data from a population-based registry that contains information regarding all births in Korea during the considered time period.

In conclusion, we observed an increase in the incidence of GDM during the short study period, especially mild GDM among Korean women. Although we observed changes in several risk factors related to GDM, the exact factors contributing to these trends were not elucidated. Further efforts are needed to monitor these trends and to identify the associated factors.

## References

[1] Ferrara A. Increasing prevalence of gestational diabetes mellitus: a public health perspective. Diabetes Care. 2007; 30 Suppl 2: S141–146. 10.2337/dc07-s206 . 17596462

[2] Chen Y, Quick WW, Yang W, Zhang Y, Baldwin A, Moran J, et al Cost of gestational diabetes mellitus in the United States in 2007. Popul Health Manag. 2009; 12: 165–174. 10.1089/pop.2009.12303 . 19534581

[3] Schmidt MI, Duncan BB, Reichelt AJ, Branchtein L, Matos MC, Costa e Forti A, et al Gestational diabetes mellitus diagnosed with a 2-h 75-g oral glucose tolerance test and adverse pregnancy outcomes. Diabetes Care. 2001; 24: 1151–1155. . 11423494 10.2337/diacare.24.7.1151

[4] Murgia C, Berria R, Minerba L, Malloci B, Daniele C, Zedda P, et al Gestational diabetes mellitus in Sardinia: results from an early, universal screening procedure. Diabetes Care. 2006; 29: 1713–1714. 10.2337/dc06-0635 . 16801613

[5] Davenport MH, Campbell MK, Mottola MF. Increased incidence of glucose disorders during pregnancy is not explained by pre-pregnancy obesity in London, Canada. BMC Pregnancy Childbirth. 2010; 10: 85 10.1186/1471-2393-10-85 . 21184681 PMC3022738

[6] Albrecht SS, Kuklina EV, Bansil P, Jamieson DJ, Whiteman MK, Kourtis AP, et al Diabetes trends among delivery hospitalizations in the U.S., 1994–2004. Diabetes Care. 2010; 33: 768–773. 10.2337/dc09-1801 . 20067968 PMC2845025

[7] Ferrara A, Kahn HS, Quesenberry CP, Riley C, Hedderson MM. An increase in the incidence of gestational diabetes mellitus: Northern California, 1991–2000. Obstet Gynecol. 2004; 103: 526–533. 10.1097/01.AOG.0000113623.18286.20 . 14990417

[8] Dabelea D, Snell-Bergeon JK, Hartsfield CL, Bischoff KJ, Hamman RF, McDuffie RS, et al Increasing prevalence of gestational diabetes mellitus (GDM) over time and by birth cohort: Kaiser Permanente of Colorado GDM Screening Program. Diabetes Care. 2005; 28: 579–584. . 15735191 10.2337/diacare.28.3.579

[9] Anna V, van der Ploeg HP, Cheung NW, Huxley RR, Bauman AE. Sociodemographic correlates of the increasing trend in prevalence of gestational diabetes mellitus in a large population of women between 1995 and 2005. Diabetes Care. 2008; 31: 2288–2293. 10.2337/dc08-1038 . 18809630 PMC2584183

[10] Sella T, Shalev V, Elchalal U, Chovel-Sella A, Chodick G. Screening for gestational diabetes in the 21st century: a population-based cohort study in Israel. J Matern Fetal Neonatal Med. 2013; 26: 412–416. 10.3109/14767058.2012.733761 . 23035769

[11] Zhang F, Dong L, Zhang CP, Li B, Wen J, Gao W, et al Increasing prevalence of gestational diabetes mellitus in Chinese women from 1999 to 2008. Diabet Med. 2011; 28: 652–657. 10.1111/j.1464-5491.2010.03205.x . 21569085

[12] Chu SY, Callaghan WM, Kim SY, Schmid CH, Lau J, England LJ, et al Maternal obesity and risk of gestational diabetes mellitus. Diabetes Care. 2007; 30: 2070–2076. 10.2337/dc06-2559a . 17416786

[13] Ben-Haroush A, Yogev Y, Hod M. Epidemiology of gestational diabetes mellitus and its association with Type 2 diabetes. Diabet Med. 2004; 21: 103–113. . 14984444 10.1046/j.1464-5491.2003.00985.x

[14] Savitz DA, Janevic TM, Engel SM, Kaufman JS, Herring AH. Ethnicity and gestational diabetes in New York City, 1995–2003. BJOG. 2008; 115: 969–978. 10.1111/j.1471-0528.2008.01763.x . 18651880

[15] Chu SY, Abe K, Hall LR, Kim SY, Njoroge T, Qin C. Gestational diabetes mellitus: all Asians are not alike. Prev Med. 2009; 49: 265–268. 10.1016/j.ypmed.2009.07.001 . 19596364

[16] White P. Pregnancy complicating diabetes. Am J Med. 1949; 7: 609–616. . 15396063 10.1016/0002-9343(49)90382-4

[17] Sendag F, Terek MC, Itil IM, Oztekin K, Bilgin O. Maternal and perinatal outcomes in women with gestational diabetes mellitus as compared to nondiabetic controls. J Reprod Med. 2001; 46: 1057–1062. . 11789086

[18] Lee AJ, Hiscock RJ, Wein P, Walker SP, Permezel M. Gestational diabetes mellitus: clinical predictors and long-term risk of developing type 2 diabetes: a retrospective cohort study using survival analysis. Diabetes Care. 2007; 30: 878–883. 10.2337/dc06-1816 . 17392549

[19] Choi NK, Chang Y, Choi YK, Hahn S, Park BJ. Signal detection of rosuvastatin compared to other statins: data-mining study using national health insurance claims database. Pharmacoepidemiol Drug Saf. 2010; 19: 238–246. 10.1002/pds.1902 . 20049851

[20] American College of Obstetricians and Gynecologists Committee on Practice Bulletins—Obstetrics. ACOG Practice Bulletin. Clinical management guidelines for obstetrician-gynecologists. Number 30, September 2001 (replaces Technical Bulletin Number 200, December 1994). Gestational diabetes. Obstet Gynecol. 2001; 98: 525–538. . 11547793

[21] Chon SJ, Choi YR, Roh YH, Yun BH, Cho S, Choi YS, et al Association between levels of serum ferritin and bone mineral density in Korean premenopausal and postmenopausal women: KNHANES 2008–2010. PLoS One. 2014; 9: e114972 10.1371/journal.pone.0114972 . 25522357 PMC4270774

[22] Oh SW, Shin SA, Yun YH, Yoo T, Huh BY. Cut-off point of BMI and obesity-related comorbidities and mortality in middle-aged Koreans. Obes Res. 2004; 12: 2031–2040. 10.1038/oby.2004.254 . 15687405

[23] Lee SY, Park HS, Kim DJ, Han JH, Kim SM, Cho GJ, et al Appropriate waist circumference cutoff points for central obesity in Korean adults. Diabetes Res Clin Pract. 2007; 75: 72–80. 10.1016/j.diabres.2006.04.013 . 16735075

[24] Expert Panel on Detection Evaluation Treatment of High Blood Cholesterol in Adults. Executive Summary of The Third Report of The National Cholesterol Education Program (NCEP) Expert Panel on Detection, Evaluation, And Treatment of High Blood Cholesterol In Adults (Adult Treatment Panel III). JAMA. 2001; 285: 2486–2497. . 11368702 10.1001/jama.285.19.2486

[25] Genuth S, Alberti KG, Bennett P, Buse J, Defronzo R, Kahn R, et al Follow-up report on the diagnosis of diabetes mellitus. Diabetes Care. 2003; 26: 3160–3167. . 14578255 10.2337/diacare.26.11.3160

[26] Kim SY, Dietz PM, England L, Morrow B, Callaghan WM. Trends in pre-pregnancy obesity in nine states, 1993–2003. Obesity (Silver Spring). 2007; 15: 986–993. 10.1038/oby.2007.621 . 17426334

[27] Jang HC, Cho NH, Jung KB, Oh KS, Dooley SL, Metzger BE. Screening for gestational diabetes mellitus in Korea. Int J Gynaecol Obstet. 1995; 51: 115–122. . 8635631 10.1016/0020-7292(95)02524-g

[28] Choi SK, Park IY, Shin JC. The effects of pre-pregnancy body mass index and gestational weight gain on perinatal outcomes in Korean women: a retrospective cohort study. Reprod Biol Endocrinol. 2011; 9: 6 10.1186/1477-7827-9-6 . 21241516 PMC3033321

[29] Rhee SY, Park SW, Kim DJ, Woo J. Gender disparity in the secular trends for obesity prevalence in Korea: analyses based on the KNHANES 1998–2009. Korean J Intern Med. 2013; 28: 29–34. 10.3904/kjim.2013.28.1.29 . 23345994 PMC3543958

[30] Dempsey JC, Sorensen TK, Williams MA, Lee IM, Miller RS, Dashow EE, et al Prospective study of gestational diabetes mellitus risk in relation to maternal recreational physical activity before and during pregnancy. Am J Epidemiol. 2004; 159: 663–670. . 15033644 10.1093/aje/kwh091

[31] Radesky JS, Oken E, Rifas-Shiman SL, Kleinman KP, Rich-Edwards JW, Gillman MW. Diet during early pregnancy and development of gestational diabetes. Paediatr Perinat Epidemiol. 2008; 22: 47–59. 10.1111/j.1365-3016.2007.00899.x . 18173784 PMC2650816

[32] England LJ, Levine RJ, Qian C, Soule LM, Schisterman EF, Yu KF, et al Glucose tolerance and risk of gestational diabetes mellitus in nulliparous women who smoke during pregnancy. Am J Epidemiol. 2004; 160: 1205–1213. 10.1093/aje/ . 15583373

[33] Park S, Kim MY, Baik SH, Woo JT, Kwon YJ, Daily JW, et al Gestational diabetes is associated with high energy and saturated fat intakes and with low plasma visfatin and adiponectin levels independent of prepregnancy BMI. Eur J Clin Nutr. 2013; 67: 196–201. 10.1038/ejcn.2012.207 . 23385969

[34] Metzger BE, Coustan DR. Summary and recommendations of the Fourth International Workshop-Conference on Gestational Diabetes Mellitus. The Organizing Committee. Diabetes Care. 1998; 21 Suppl 2: B161–167. . 9704245

[35] American Diabetes Association. Gestational diabetes mellitus. Diabetes Care. 2002; 25 (Suppl 1): S94–96. 10.2337/diacare.25.2007.s1 11788484

[36] Cheng YW, Block-Kurbisch I, Caughey AB. Carpenter-Coustan criteria compared with the national diabetes data group thresholds for gestational diabetes mellitus. Obstet Gynecol. 2009; 114: 326–332. 10.1097/AOG.0b013e3181ae8d85 . 19622994

[37] Ferrara A, Hedderson MM, Quesenberry CP, Selby JV. Prevalence of gestational diabetes mellitus detected by the national diabetes data group or the carpenter and coustan plasma glucose thresholds. Diabetes Care. 2002; 25: 1625–1630. . 12196438 10.2337/diacare.25.9.1625

[38] Ricart W, López J, Mozas J, Pericot A, Sancho MA, González N, et al Potential impact of American Diabetes Association (2000) criteria for diagnosis of gestational diabetes mellitus in Spain. Diabetologia. 2005; 48: 1135–1141. 10.1007/s00125-005-1756-9 . 15889233

[39] Wong VW, Jalaludin B. Gestational diabetes mellitus: who requires insulin therapy? Aust N Z J Obstet Gynaecol. 2011; 51: 432–436. 10.1111/j.1479-828X.2011.01329.x . 21806589

[40] Crowther CA, Hiller JE, Moss JR, McPhee AJ, Jeffries WS, Robinson JS, et al Effect of treatment of gestational diabetes mellitus on pregnancy outcomes. N Engl J Med. 2005; 352: 2477–2486. 10.1056/NEJMoa042973 . 15951574

[41] Wong VW. Gestational diabetes mellitus in five ethnic groups: a comparison of their clinical characteristics. Diabet Med. 2012; 29: 366–371. 10.1111/j.1464-5491.2011.03439.x . 21913963

[42] Kim JY, Kim BO, Kang DH, Bae HJ, Kim HC, Kim MS, et al Construction of national surveillance system for cardiovascular & cerebrovascular diseases Seoul: Health Insurance Review & Assessment Service 2006.

